# Evaluating International Diagnostic, Screening, and Monitoring Practices for Craniofacial Microsomia and Microtia: A Survey Study

**DOI:** 10.1177/10556656221093912

**Published:** 2022-04-26

**Authors:** Elsa M. Ronde, Jitske W. Nolte, Frea H. Kruisinga, Saskia M. Maas, Oren Lapid, Fenna A. Ebbens, Alfred G. Becking, Corstiaan C. Breugem

**Affiliations:** 1 Amsterdam UMC location University of Amsterdam, Plastic, Reconstructive and Hand Surgery, Amsterdam, the Netherlands; 2 Amsterdam UMC location University of Amsterdam, Oral and Maxillofacial Surgery, Amsterdam, the Netherlands; 3 Amsterdam Reproduction and Development, Amsterdam, the Netherlands; 4 Amsterdam Movement Sciences, Amsterdam, the Netherlands; 5 Amsterdam UMC location University of Amsterdam, Pediatrics, Amsterdam, the Netherlands; 6 Amsterdam UMC location University of Amsterdam, Clinical Genetics, Amsterdam, the Netherlands; 7 Amsterdam UMC location University of Amsterdam, Otorhinolaryngology, Amsterdam, the Netherlands; 8 Amsterdam Public Health, Ear and Hearing, Amsterdam, the Netherlands

**Keywords:** craniofacial microsomia, microtia, diagnostics, screening, clinical management, clinician survey

## Abstract

**Objectives:**

To (1) appraise current international classification and clinical management strategies for craniofacial microsomia (CFM) and microtia, and (2) to assess agreement with the European Reference Network “European Guideline Craniofacial Microsomia” recommendations on screening and monitoring.

**Design:**

This was a cross-sectional online survey study. The survey consisted of 44 questions on demographics, diagnostics and classification, obstructive sleep apnea, feeding difficulties, speech and language development, hearing, ocular abnormalities, visual development, orthodontic screening, genetic counselling, psychological wellbeing, and extracraniofacial anomalies.

**Participants:**

Respondents were participants of 3 international cleft and craniofacial conferences, members of the American Cleft Palate and Craniofacial Association and members of the International Society for Auricular Reconstruction. Respondents were requested to complete 1 questionnaire per multidisciplinary team.

**Results:**

Fifty-seven responses were received from 30 countries (response rate ∼3%).The International Consortium for Health Outcomes Measurement diagnostic criteria were used by 86% of respondents, though 65% considered isolated microtia a mild form of CFM. The Orbit, Mandible, Ear, Facial Nerve and Soft Tissue classification system was used by 74% of respondents. Agreement with standardized screening and monitoring recommendations was between 61% and 97%. A majority of respondents agreed with screening for extracraniofacial anomalies (63%-68%) and with genetic counselling (81%).

**Conclusions:**

This survey did not reveal consistent agreement on the diagnostic criteria for CFM. Respondents mostly supported management recommendations, but frequently disagreed with the standardization of care. Future studies could focus on working towards international consensus on diagnostic criteria, and exploring internationally feasible management strategies.

## Introduction

Craniofacial microsomia (CFM) is characterized by hypoplasia of the craniofacial structures and ears, or microtia, and is estimated to occur in 1:5500 to 1:26000 live births.^1–3^ Diagnosing and managing patients with CFM is challenging due to the phenotypical heterogeneity associated with the condition and the lack of consensus on the inclusion of isolated microtia within the phenotypic spectrum.

Recently, the European Reference Network (ERN) CRANIO Working Group on Craniofacial Microsomia proposed the European Guideline Craniofacial Microsomia, a first international guideline for CFM.^4^ The ERN guideline was developed by a steering group consisting of 4 maxillofacial surgeons, 3 plastic surgeons, and a maxillofacial surgery research fellow, who consulted 9 additional experts from various fields for review and recommendations. In the ERN guideline, the diagnosis of CFM is defined according to criteria developed by the International Consortium for Health Outcomes Measurement (ICHOM). According to these criteria, isolated microtia is not considered a mild form of CFM.^5^ The ERN guideline details recommendations for diagnostics, screening, monitoring, and treatment, and due to the lack of applicable literature, many recommendations regarding diagnostics and screening are based on the expert opinion of the ERN guideline authors. Furthermore, the ERN guideline does not include recommendations on classification systems, craniofacial radiographic imaging, genetic counselling or screening for extracraniofacial anomalies besides vertebral anomalies.

The aim of this study is to (1) appraise current international classification and clinical management strategies for CFM and microtia, and (2) to assess international agreement with the ERN guideline's recommendations pertaining to the screening and monitoring of patients with CFM.

## Materials and Methods

The Medical Ethics Review committee of the Academic Medical Center (Amsterdam UMC) waived the need for full ethical board review for this cross-sectional survey study (W21_318 # 21.353). The Checklist for Reporting Of Survey Studies (CROSS) was followed^6^ (Appendix A).

### Survey Design

Survey questions consisted of a combination of the existing ERN guideline recommendations on screening and monitoring, and additional questions based on the authors’ views of perceived knowledge gaps and topics not covered by the ERN guideline. Author-written questions covered demographics, the inclusion of isolated microtia within the CFM spectrum, the management of isolated microtia, the classification of patients and craniofacial imaging, screening for extracraniofacial anomalies and genetic screening. Survey questions on ERN guideline recommendations were explicitly cited. Survey questions are available in Appendix B. The survey included 44 multiple choice-, checkbox- and open-ended questions with comment fields for all multiple choice- and checkbox questions. Multiple choice questions were either formatted as statements on a 3-point Likert scale (ie “agree,” “disagree,” and “none of the above”), or as questions with discrete answer options. Respondents were requested to clarify their response in the comment field in case they did not agree with the statement or wished to comment. Additionally, a comment field was provided for feedback on the survey. All parts of the survey and the invitation were in English.

### Survey Distribution

The survey was distributed using SurveyMonkey Inc., an online survey software.^7^ The intended respondents were multidisciplinary teams (MDT) providing care for patients with CFM and microtia care. Simple random sampling was applied by sending survey invitations directly to all participants of the 2015 European Cleft Craniofacial congress in Gothenburg, Sweden, the 2017 13th International Cleft congress in Chennai, India, and the 2019 European Cleft Palate Craniofacial Association congress in Utrecht, the Netherlands in February 2022 using up-to-date and deduplicated mailing lists with consent. A reminder was sent 3 weeks after the initial invitation. Furthermore, a link to the survey was placed in the member portal of the American Cleft Palate—Craniofacial Association (ACPA) and was shared with members of the International Society for Auricular Reconstruction (ISAR) through the WhatsApp group for all members. These methods were chosen for a broad global distribution of the survey. Respondents were requested to answer the questions according to the clinical pathway of the MDT at their hospital, if applicable, and to provide 1 answer per MDT, if possible.

### Data Analysis

The data are presented as the number (N) and proportion (%) of respondents. The data were collected and analyzed confidentially using SPSS (v27.0, IBM Corp, Armonk, NY, USA).^8^ Questions were analyzed individually and missing answers were documented. Differences in responses were examined according to geographical location and discipline using the Fisher's exact test, provided that the sample size for subgroups was 5 or more.

## Results

### Demographics

The survey was directly sent to 2086 clinicians, and 57 responses were received (response rate ∼3%). Most respondents were plastic surgeons, maxillofacial surgeons or orthodontists (Table 1). Most of the respondents (60%) were based in Europe. A total of 30 countries and 6 continents were represented (Supplemental table 1).

**Table 1.** Respondent Characteristics (N    =    57).

Characteristic	Number of respondents (%)
Discipline^a^	
Plastic surgeon	24 (42)
Oral and maxillofacial surgeon	15 (26)
Orthodontist	10 (18)
ENT surgeon/otorhinolaryngologist	3 (5.3)
Speech and language therapist	3 (5.3)
Pediatrician	2 (3.5)
Clinical geneticist	2 (3.5)
Neurosurgeon	1 (1.8)
Pediatric surgeon	1 (1.8)
Multidisciplinary team (not further specified)	1 (1.8)
Number of disciplines checked by respondent	1 (1.8)
0^b^	1 (1.8)
1	51 (89)
2	5 (8.8)
Location	
Europe	34 (60)
Asia	11 (19)
North America	6 (11)
South America	3 (5.3)
Oceania	2 (2.5)
Africa	1 (1.8)

ENT: Ear-, Nose-, and Throat.

^a^ 5 respondents checked multiple disciplines: plastic surgeon and oral and maxillofacial surgeon (3); ENT surgeon and speech and language therapist (1); oral and maxillofacial surgeon and orthodontist (1).

^b^ Responded as representative of the team.

Five questionnaires (9%) were incomplete. Questions with missing responses and the number of missing responses are reported in Table 2 which presents answers to multiple choice questions. Checkbox questions and open-ended questions were answered by all respondents. Qualitative comments are provided in Supplemental table 2.

**Table 2.** Multiple Choice Questions (N = 57).

Question number	Question summary	Agree	Disagree	Neither agree nor disagree	Not answered^b^
5	ICHOM criteria for CFM^a^ (3.1)	49 (86)	6 (11)	2 (3.5)	-
6	Isolated microtia is a mild form of CFM	37 (65)	19 (33)	1 (1.8)	-
11	Biannual screening for obstructive sleep apnea at least until age of 6 years^a^ (4.1.2)	37 (65)	14 (25)	6 (11)	-
12	Polysomnography in case of OSA suspicion based on questionnaire^a^ (4.1.2)	54 (95)	2 (3.5)	1 (1.8)	-
13	Polysomnography for all Pruzansky-Kaban IIa or III or bilaterally affected mandibles^a^ (4.1.2)	38 (67)	15 (26)	4 (7.0)	-
14	Biannual screening for feeding difficulties at least until 6 years^a^ (4.2.2)	37 (65)	15 (26)	5 (8.8)	-
15	Screening for preverbal communication at age 9 months^a^ (4.3.2)	35 (61)	14 (25)	6 (11)	2 (3.5)
16	Biannual language evaluation between 2 and 8 years^a^ (4.3.2)	43 (75)	11 (19)	3 (5.3)	-
17	In patients with cleft palate: annual screening between 2 and 5 years by specialized speech and language therapist and adherence to cleft palate protocol^a^ (4.3.2)	52 (91)	4 (7.0)	1 (1.8)	-
18	In patients with cleft palate: VPD assessment at 2 years or emergence of verbal output^a^ (4.3.2)	44 (77)	10 (18)	3 (5.3)	-
19	VPD screening at age 2 years in all patients^a^ (4.3.2)	40 (70)	14 (25)	3 (5.3)	-
20	Neonatal hearing test in all patients^a^ (4.4.2)	53 (93)	4 (7/0)	-	-
21	Complete audiological evaluation before age 3 months (if indicated)^a^ (4.4.2)	55 (96)	2 (3.5)	-	-
22	Independent hearing assessment next to any national assessment	45 (79)	7 (12)	4 (7.0)	1 (1.8)
23	Screening by orthoptist and ophthalmologist before age 5 years^a^ (4.5.2)	50 (88)	5 (8.8)	2 (7.0)	-
24	Referral to ophthalmologist in case of lagophthalmos^a^ (5.2.1)	52 (91)	3 (5.3)	1 (1.8)	1 (1.8)
25	Cleft Q appearance for assessment of facial movement at ages 8, 12, and 22 years^a^ (5.2.1)	44 (77)	9 (16)	4 (7.0)	-
26	Screening by orthodontist from age 5 years for dental deformities^a^ (4.6.2)	53 (93)	3 (5.3)	1 (1.8)	-
27	Orthodontic records at ages 6, 9, 12, 15, and 18 years^a^ (4.6.2)	42 (74)	10 (18)	4 (7.0)	1 (1.8)
28	Screening for neck/back symptoms during initial consultation and preoperatively^a^ (4.7.2)	48 (84)	4 (7.0)	4 (7.0)	1 (1.8)
29	Neurological evaluation in case of neurologic symptoms (neck/back)^a^ (4.7.2)	53 (93)	2 (3.5)	1 (1.9)	1 (1.8)^b^
30	Spine radiography for vertebral screening in all patients	36 (63)	15 (26)	5 (8.8)	1 (1.8)
32	Echocardiography for cardiac screening in all patients	36 (63)	15 (26)	4 (7.0)	2 (4)
34	Renal ultrasonography for renal screening in all patients	39 (68)	11 (19)	5 (8.8)	2 (3.5)
36	Screening for additional anomalies (not mentioned above) for all patients	27 (47)	23 (40)	4 (7.0)	3 (5.3)
37	Screening by clinical geneticist for all patients	46 (81)	8 (14)	1 (1.8)	2 (3.5)
39	Access to clinical psychology service (all patients)^a^ (4.8.2)	50 (88)	4 (7.0)	2 (3.5)	1 (1.8)
40	Screening for psychological wellbeing at key life transitions^a^ (4.8.2)	53 (93)	2 (3.5)	1 (1.8)	1 (1.8)
41	Routinely screening for psychosocial wellbeing and family stress using validated self-reported psychological outcome measures at ages 2, 5, 8 and 22^a^ (4.8.2)	41 (72)	12 (21)	3 (5.3)	1 (1.8)
43	Same diagnostic and screening protocol for patients with isolated microtia	25 (44)	29 (51)	3 (5.3)	-

Abbreviations: ICHOM, International Consortium of Health Outcomes Measurement; CFM, craniofacial microsomia; OSA, obstructive sleep apnea; VPD, velopharyngeal dysfunction.

^a^ From European Guideline Craniofacial Microsomia (chapter).

^b^ One answer was considered missing/not answered due to a clear misinterpretation of the question (Supplemental table 2, question 29).

### General Diagnostics and Classification

**Figure 1.**
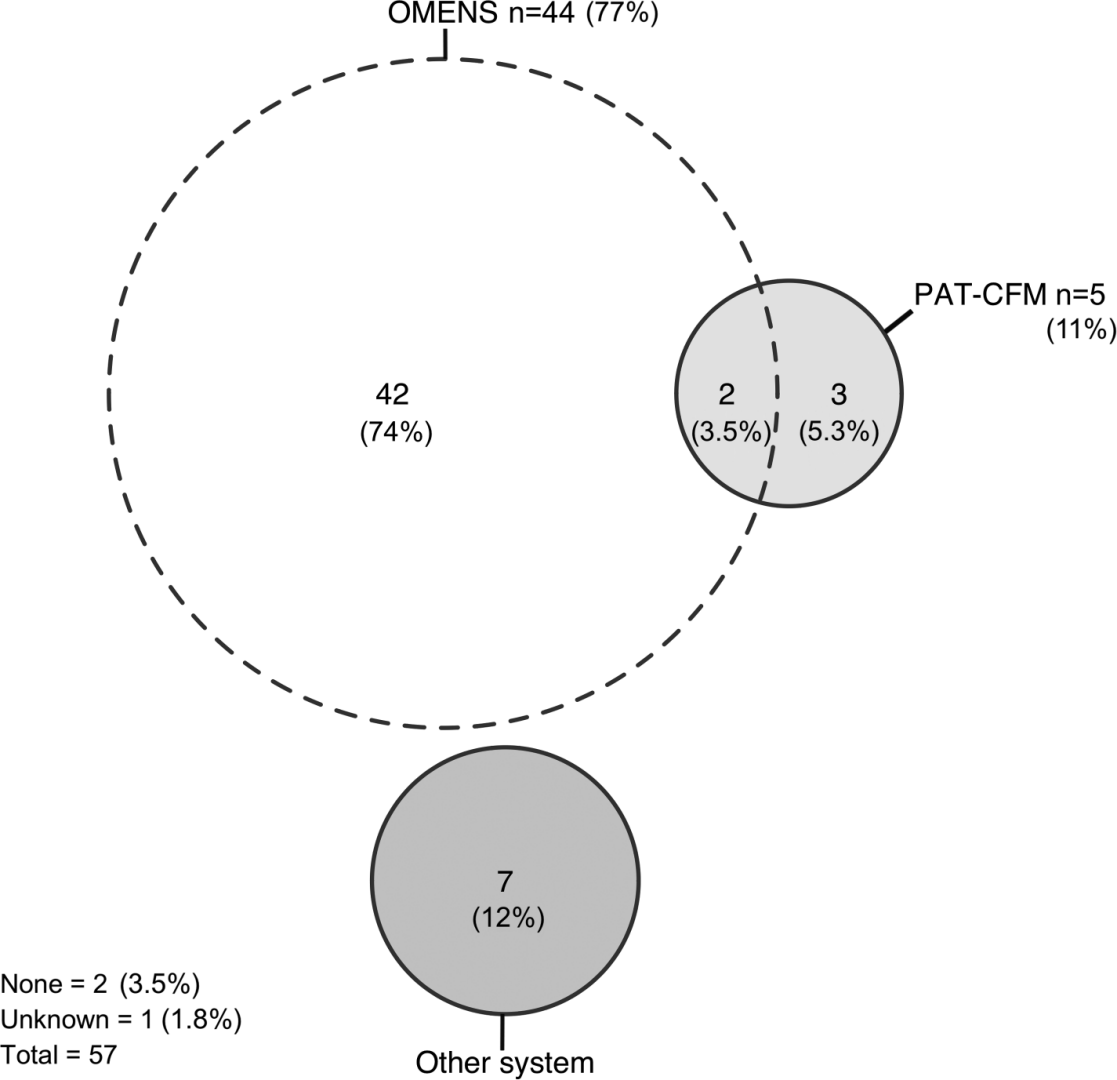
Euler diagram illustrating classification systems used by respondents. Each circle represents one answer option. Sections where 2 or more circles overlap indicate that respondents checked multiple answer options. The results are presented as the number (n) and proportion (%) of respondents.

Abbreviations: OMENS, Orbit, Mandible, Ear, (Facial) Nerve and Soft tissue classification system; PAT-CFM, phenotypic assessment tool—craniofacial microsomia.

**Figure 2.**
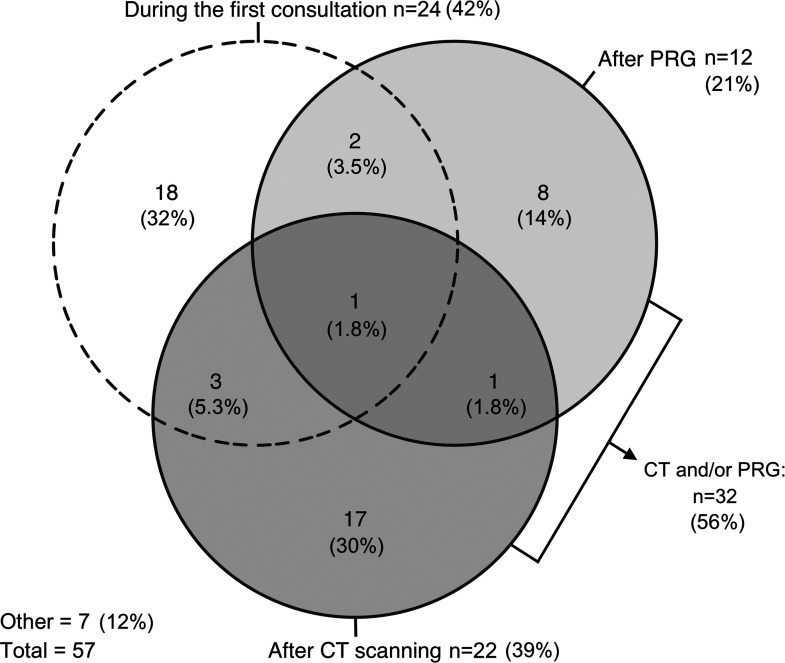
Euler diagram illustrating respondents’ preferences for definitively classifying patients. Each circle represents one answer option. Sections where 2 or more circles overlap indicate that respondents checked multiple answer options. The results are presented as the number (n) and proportion (%) of respondents.

Abbreviations: PRG, panoramic radiography; CT, computed tomography.

**Figure 3.**
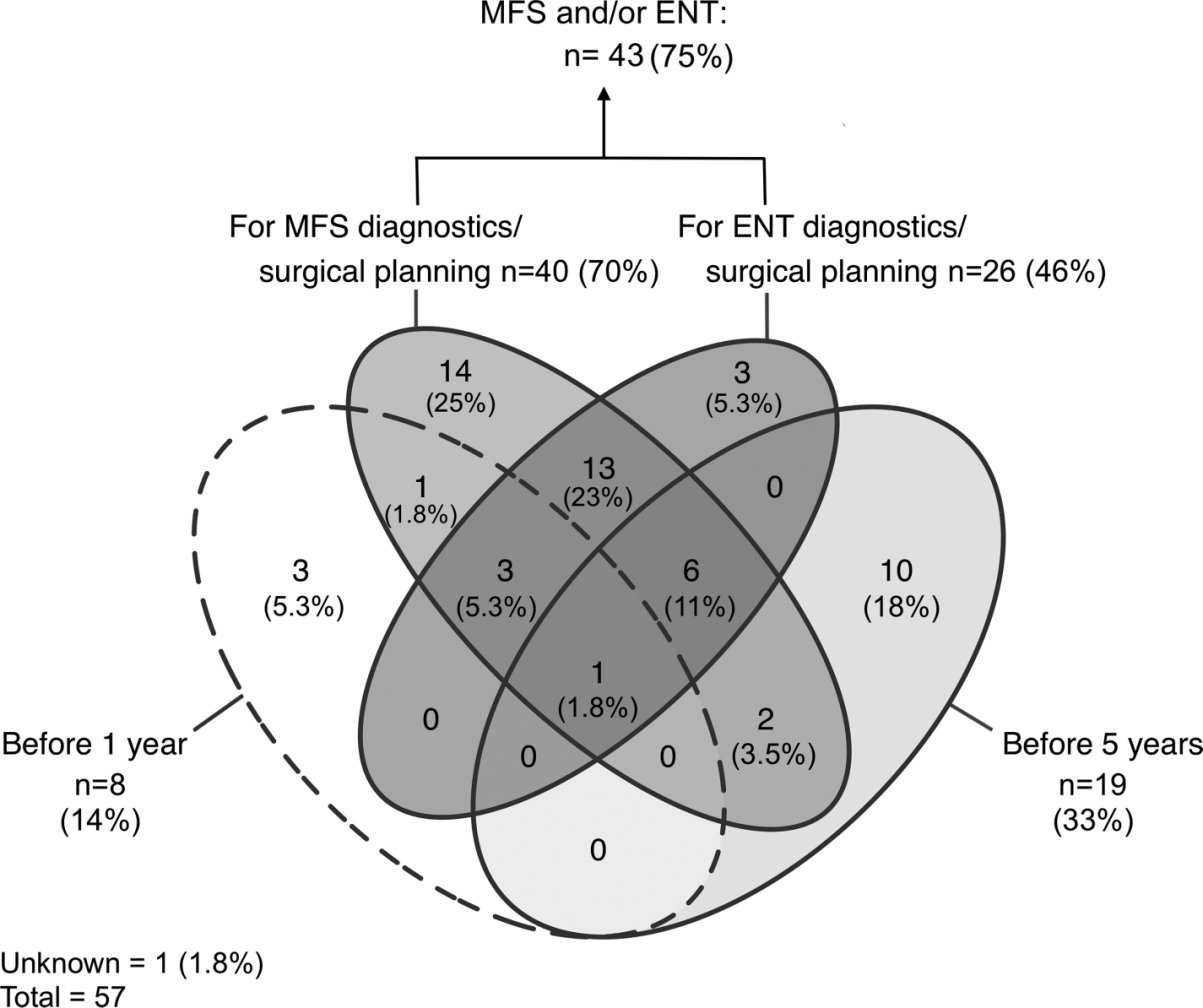
Venn diagram illustrating respondents’ preferences for scheduling CT imaging. Each ellipse represents one answer option. Sections where 2 or more ellipses overlap indicate that respondents checked multiple answer options. The results are presented as the number (n) and proportion (%) of respondents.

Abbreviations: MFS, maxillofacial surgery; ENT, Ear-, nose-, and throat surgery (otorhinolaryngology).

A majority of respondents (86%) agreed with the ICHOM criteria for classifying CFM, although 65% of respondents also considered isolated microtia a mild form of CFM (Table 2). The Orbit, Mandible, Ear, Facial Nerve and Soft tissue (OMENS)^9^ classification system was most frequently (n    =    44, 77%) used for classifying patients (Figure 1). The majority of respondents (n    =    32, 56%) definitively classified patients after computed tomography (CT), panoramic radiography (PRG) or both (Figure 2). Most respondents (n    =    43, 75%) preferred scheduling CT scanning when required for diagnostics or surgical planning for maxillofacial surgery, ear, nose and throat (ENT) surgery/otorhinolaryngology or both (Figure 3). Respondents thought CT scanning should be used to evaluate at least the mandible (96%), maxilla (84%), the zygomatic- (84%) and temporal bones (84%), and the orbits (63%) (Supplemental figure 1).

Half of respondents (51%) would not apply the entire CFM diagnostic, screening and monitoring protocol for patients with isolated microtia (Table 2). Four respondents considered this workup necessary in order to diagnose microtia as an isolated anomaly (Supplemental table 2). Agreement on which disciplines are needed in a craniofacial team treating patients with CFM was 32% to 100% (Supplemental figure 2).

### Screening for OSA and Feeding Difficulties

A majority (65%) of respondents agreed with biannual screening for OSA and feeding difficulties, and 95% considered polysomnography necessary in patients with suspected OSA, or Pruzansky-Kaban IIb, III or bilaterally affected mandibles (67%) (Table 2). Reasons for disagreement were most frequently related to the recommendation to screen all patients or the suggested screening frequency (Supplemental table 2).

### Orthodontic Screening

Most respondents (93%) concurred on orthodontic screening for dental and skeletal deformities (74%) (Table 2). Four respondents (7%); 2 maxillofacial surgeons and 2 orthodontists, indicated that records should start at age 5 years, instead of 6 years as proposed in the ERN guideline (Supplemental table 2).

### Audiological Assessment and Assessment of Language and Speech Development

Nearly all respondents agreed with the recommendations on neonatal audiological evaluation and adherence to the local cleft palate protocol in cases of concurrent cleft palate (91%-97%). The majority (61%-75%) also agreed with recommendations relating to speech and language assessment (Table 2). Reasons for disagreement were most frequently related to the timing of screening, the frequency of screening or the recommendation to screen all patients (Supplemental table 2).

### Screening for Ocular Anomalies, Assessment of Visual Development and Facial Nerve Function

Most respondents (88%) agreed with ocular and visual screening, referring patients with lagophthalmos to an ophthalmologist (91%) and assessing facial movement with the CleftQ (77%) (Table 2). Four disagreeing respondents disagreed with the use of the CleftQ (Supplemental table 2).

### Screening for ECFA and Assessment by Clinical Geneticist

**Figure 4.**
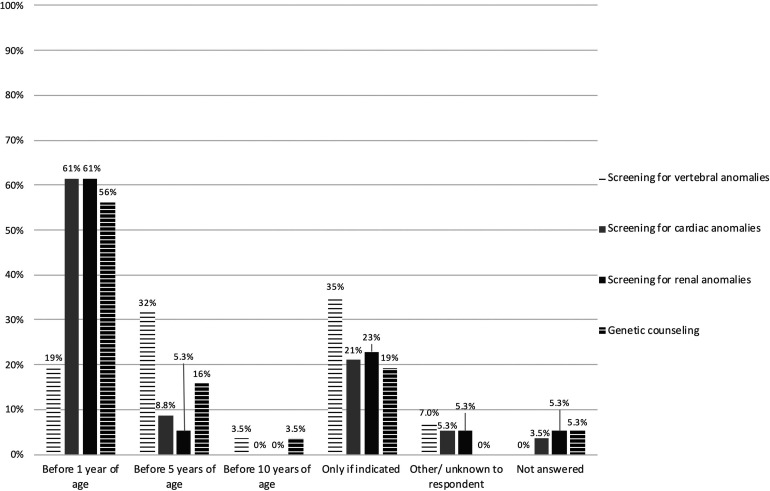
Bar chart illustrating respondents’ preferences for scheduling screening for extracraniofacial anomalies and genetic counseling. Results are presented as proportions (%) of respondents that checked an answer option. N    =    57.

A large majority of respondents agreed with screening for neck and back symptoms (84%) and complete neurological evaluation in case of neurological symptoms (93%). Most respondents also agreed with screening for vertebral, cardiac, and renal anomalies using spinal radiography (63%), echocardiography (63%), and renal ultrasonography (68%) (Table 2). Disagreements related to the standardized screening of all patients (Supplemental table 2). Most respondents (61%) indicated that screening for renal and cardiac anomalies should be done before the age of 1 year (Figure 4). No clear consensus was reached on the timing of vertebral screening (Figure 4). Furthermore, 81% considered genetic counselling important in all patients (Table 2), and 56% of respondents preferred genetic counselling within the first year of life (Figure 4).

### Assessment of Psychological Wellbeing

Most respondents agreed with the recommendations for access to a psychology service and routine psychological screening (Table 2). Disagreements were generally related to the proposed questionnaires or the proposed screening frequency.

### Analyses

Responses to multiple-choice questions did not vary significantly (*P* > .05) between plastic surgeons (n    =    21), oral and maxillofacial surgeons (n    =    15), and orthodontists (n    =    10). Intercontinental variation between Asia, Europe, and North America was significant (*P*    =    .037) for question 18 (“*Velopharyngeal dysfunction should be assessed at the age of two years or when verbal output has emerged”)*.^4^ *A*ll respondents from North America agreed (n    =    6), compared to 85% of European (29/34) and 45% of Asian respondents (5/11). Four Asian respondents clarified their disagreement: 3 preferred assessment of velopharyngeal dysfunction around 3 or 4 years of age and one at 1 year of age.

## Discussion

Management strategies for CFM and microtia are difficult to standardize due to the range in phenotypic presentation and symptom severity. The literature is heterogeneous and commonly retrospective in nature, restricting management strategies largely to expert opinion. Though recently efforts have been made by the ICHOM and ERN CRANIO working groups to standardize clinical management, to our knowledge, no large-scale international consensus exists on screening and monitoring protocols.

Various minimal diagnostic criteria for CFM are used. These generally comprise of mandibular hypoplasia,^10–12^ microtia,^13–15^ or a combination of at least 2 anomalies associated with CFM.^3,16,17^ Whether isolated microtia can be considered the mildest form of CFM is debated in particular.^18^ Proponents’ arguments generally include the phenotypical similarity of the ear malformations,^19^ the higher incidence of isolated microtia, preauricular tags, and preauricular pits in families of patients with CFM,^14,15,20^ the occurrence of extracraniofacial anomalies in patients with isolated microtia,^13–15,21–23^ and the corresponding etiological hypotheses.^24,25^ The ICHOM criteria exclude isolated microtia from the phenotypic spectrum of CFM. These criteria define CFM by the presence of: (1) 2 major criteria; (2) 1 major and 1 minor criterion; or (3) 3 or more minor criteria.^5^ Most of the ICHOM major criteria correspond to the craniofacial expressions of CFM that are generally considered characteristic to the condition (ie, mandibular hypoplasia, microtia, orbital hypoplasia, facial soft tissue hypoplasia, and facial nerve involvement).^9,26,27^ However, the minor criteria only include a limited selection of other anomalies associated with CFM, excluding other craniofacial anomalies such as coloboma,^15,27,28^ soft palate dysfunction, and velopharyngeal insufficiency.^29,30^ Furthermore, the minor criteria comprise of specific anomalies (eg, hemivertebrae) instead of general descriptors (eg, vertebral anomalies). The ICHOM working group should consider providing more information regarding how the diagnostic criteria were drafted, as to our knowledge, these details have not been published. In this survey study, no clear consensus was reached, as a majority agreed with the ICHOM criteria and contrastingly also considered isolated microtia a mild form of CFM.

Although a majority of respondents agreed on most of the questions included in this survey, agreement was <75% for half of the survey questions, a commonly used threshold for consensus.^31^ Respondents frequently disagreed on standardized screening and monitoring, preferring individualized management severity of the condition or the presence of relevant symptoms. Individualizing treatment by severity is limited by the lack of high quality literature evidence, as well as largely subjective severity scores such as the OMENS classification. Recently, a large retrospective cohort study identified a higher incidence of feeding difficulties, OSA and extracraniofacial anomalies in patients with more severe mandibular hypoplasia, as defined by the Pruzansky-Kaban criteria.^27,32,33^ Accurately assessing and grading mandibular hypoplasia according to the Pruzansky-Kaban criteria requires radiographic assessment. In the current study, clinicians preferred CT scanning over PRG for classifying patients with CFM, and only 14% of clinicians performed CT scanning in patients before the age of 1. As clinicians may consequently be reliant on subjective clinical assessment for disease severity in young patients, individualizing management of patients based on severity may be challenging. More research and international dialogue is needed to determine when radiographic assessment for disease classification should be scheduled. Future research could also focus on the reliability of subjective severity assessments, as well as objective non-ionizing forms of imaging such as stereophotogrammetry^34^ for disease classification.

The occurrence of extracraniofacial anomalies complicates the management of CFM and microtia further.^27^ To our knowledge, no consensus exists on screening protocols for extracraniofacial anomalies, and the ERN guideline only includes recommendations for screening relating to neck, back, and neurologic symptoms. The incidences of vertebral, cardiac, and renal anomalies have been reported to range between 8% and 55%,^35^ 3% and 33%^3,13,15,21,23,26,36–40^, and 3% and 20%,^3,13,15,21,22,26,37,38,40,41^ respectively, in retrospective studies of more than 50 patients. The majority of the surveyed international clinicians supported screening for at least vertebral, cardiac, and renal anomalies, largely in line with a previous reviews on management recommendations, though there was no definitive consensus.^19,42^

Several respondents from Europe, North America, and Asia indicated limitations in screening and monitoring due to costs or the lack of insurance coverage (Supplemental table 2). This raises the question of the feasibility of the widespread implementation of the ERN guideline. This survey study only found significant geographical variation in the responses of a single question, which did not reveal concerns regarding the inequity of care. However, the underrepresentation of several continents limited these assessments, and intracontinental assessment was not possible. Respondents also raised the possibility of minimum care requirements, when assessing which disciplines are needed in a CFM team (Supplemental table 2). The possible barriers in healthcare access for CFM and microtia, their effect on the implementation of international guidelines, as well as the possibility of minimum care requirements could be an important focus for future research in this population.

Assessing comprehension of ERN guideline recommendations was beyond the scope of this survey study. Several recommendations referred to “biannual” screening or monitoring, which may be interpreted as “twice a year” or “once every two years.” The corresponding author of the ERN guideline confirmed that “biannual” refers to “once every two years” in the ERN guideline.

This study has some limitations that need to be considered. The survey results may not be representative of clinical management practices across all craniofacial centers globally due to a number of factors. The response rate could not be definitively calculated, as it is conceivable that ISAR and ACPA members attended one of the included conferences and thus, may have encountered the survey in several ways. Respondents were also requested to provide 1 answer per MDT, while multiple team members may have received a survey invitation. The minimum response rate, assuming no ACPA or ISAR members attended any of the conferences is 1%. Due to the broad distribution, the sampled population may not have been fully representative of the target population. Sampling in a population restricted to healthcare providers involved in CFM or microtia care is not practical due to the lack of easily accessible and accurate global registries of centers treating these patients. Furthermore, though respondents were asked to respond according to local multidisciplinary protocols, it is uncertain whether they complied with this request, as this was not explicitly confirmed within the survey. One respondent indicated to have responded for their MDT, and 5 respondents represented more than 1 discipline, although respondents who checked multiple disciplines may also have been individuals practicing in several disciplines. Twelve non-identical responses were received from 6 hospitals, where 2 non-identical responses were received per hospital. It is unclear whether this was due to the lack of a MDT and local clinical protocols, or the lack of adherence of MDT members to clinical protocols. It cannot be ruled out that a proportion of the respondents may have described their own clinical practice.

A few other sources of bias should also be considered. Clinicians outside of Europe may have been less likely to respond due to the title of the ERN guideline, and respondents may have been more likely to agree with ERN guideline recommendations due to authority bias. Lastly, not all areas of diagnostics, screening, and monitoring of CFM could be comprehensively covered in one survey, and further international dialogue is needed for clear consensus on these topics.

This survey was not designed to reach consensus, but rather to gather first impressions of international diagnostics and evaluate agreement with the ERN guideline recommendations. These results may be a step in initiating large-scale international dialogue on the challenges involving diagnostics and management strategies. Future studies aiming for consensus could implement consensus methods such as the Delphi method,^43^ which involves iteratively surveying relevant stakeholders on a specific topic until (predefined) consensus is reached. This could be relevant for defining the minimum diagnostic criteria for CFM, but also for continued dialogue on globally acceptable management strategies.

## Conclusion

There was no clear agreement on diagnostic criteria for CFM. Clinicians supported most management recommendations, where disagreements frequently related to the standardization of care. Standardizing care for CFM and microtia is challenging due to the lack of consensus on diagnostic criteria, the varying degree of disease severity as well as potential healthcare access barriers. Future studies could focus on international consensus for diagnostic criteria, and exploring internationally feasible management strategies.

## Supplemental Material

sj-docx-1-cpc-10.1177_10556656221093912 - Supplemental material for Evaluating International Diagnostic, Screening, and Monitoring Practices for Craniofacial Microsomia and Microtia: A Survey Study Click here for additional data file. Supplemental material, sj-docx-1-cpc-10.1177_10556656221093912 for Evaluating International Diagnostic, Screening, and Monitoring Practices for Craniofacial Microsomia and Microtia: A Survey Study by Elsa M. Ronde, Jitske W. Nolte, Frea H. Kruisinga, Saskia M. Maas, Oren Lapid, Fenna A. Ebbens, Alfred G. Becking and Corstiaan C. Breugem in The Cleft Palate Craniofacial Journal

sj-docx-2-cpc-10.1177_10556656221093912 - Supplemental material for Evaluating International Diagnostic, Screening, and Monitoring Practices for Craniofacial Microsomia and Microtia: A Survey Study Click here for additional data file. Supplemental material, sj-docx-2-cpc-10.1177_10556656221093912 for Evaluating International Diagnostic, Screening, and Monitoring Practices for Craniofacial Microsomia and Microtia: A Survey Study by Elsa M. Ronde, Jitske W. Nolte, Frea H. Kruisinga, Saskia M. Maas, Oren Lapid, Fenna A. Ebbens, Alfred G. Becking and Corstiaan C. Breugem in The Cleft Palate Craniofacial Journal

sj-docx-3-cpc-10.1177_10556656221093912 - Supplemental material for Evaluating International Diagnostic, Screening, and Monitoring Practices for Craniofacial Microsomia and Microtia: A Survey Study Click here for additional data file. Supplemental material, sj-docx-3-cpc-10.1177_10556656221093912 for Evaluating International Diagnostic, Screening, and Monitoring Practices for Craniofacial Microsomia and Microtia: A Survey Study by Elsa M. Ronde, Jitske W. Nolte, Frea H. Kruisinga, Saskia M. Maas, Oren Lapid, Fenna A. Ebbens, Alfred G. Becking and Corstiaan C. Breugem in The Cleft Palate Craniofacial Journal

sj-jpg-4-cpc-10.1177_10556656221093912 - Supplemental material for Evaluating International Diagnostic, Screening, and Monitoring Practices for Craniofacial Microsomia and Microtia: A Survey Study Click here for additional data file. Supplemental material, sj-jpg-4-cpc-10.1177_10556656221093912 for Evaluating International Diagnostic, Screening, and Monitoring Practices for Craniofacial Microsomia and Microtia: A Survey Study by Elsa M. Ronde, Jitske W. Nolte, Frea H. Kruisinga, Saskia M. Maas, Oren Lapid, Fenna A. Ebbens, Alfred G. Becking and Corstiaan C. Breugem in The Cleft Palate Craniofacial Journal

sj-jpg-5-cpc-10.1177_10556656221093912 - Supplemental material for Evaluating International Diagnostic, Screening, and Monitoring Practices for Craniofacial Microsomia and Microtia: A Survey Study Click here for additional data file. Supplemental material, sj-jpg-5-cpc-10.1177_10556656221093912 for Evaluating International Diagnostic, Screening, and Monitoring Practices for Craniofacial Microsomia and Microtia: A Survey Study by Elsa M. Ronde, Jitske W. Nolte, Frea H. Kruisinga, Saskia M. Maas, Oren Lapid, Fenna A. Ebbens, Alfred G. Becking and Corstiaan C. Breugem in The Cleft Palate Craniofacial Journal

sj-docx-6-cpc-10.1177_10556656221093912 - Supplemental material for Evaluating International Diagnostic, Screening, and Monitoring Practices for Craniofacial Microsomia and Microtia: A Survey Study Click here for additional data file. Supplemental material, sj-docx-6-cpc-10.1177_10556656221093912 for Evaluating International Diagnostic, Screening, and Monitoring Practices for Craniofacial Microsomia and Microtia: A Survey Study by Elsa M. Ronde, Jitske W. Nolte, Frea H. Kruisinga, Saskia M. Maas, Oren Lapid, Fenna A. Ebbens, Alfred G. Becking and Corstiaan C. Breugem in The Cleft Palate Craniofacial Journal
