# Supplementary material for: Evaluating International Diagnostic, Screening, and Monitoring Practices for Craniofacial Microsomia and Microtia: A Survey Study
Source: Cleft Palate Craniofac J. 2022 Apr 26;60(9):1118–27. doi: 10.1177/10556656221093912 (PMC10466995; doi:10.1177/10556656221093912)
Supplement: sj-docx-2-cpc-10.1177_10556656221093912 - Supplemental material for Evaluating International Diagnostic, Screening, and Monitoring Practices for Craniofacial Microsomia and Microtia: A Survey Study [file sj-docx-2-cpc-10.1177_10556656221093912.docx]

**Supplementary table 1: Countries represented (N=57)**

| Country | Number of respondents (%) |
| --- | --- |
| Netherlands | 6 (11) |
| United States of America | 6 (11) |
| India | 5 (8.8) |
| United Kingdom | 5 (8.8) |
| Czech Republic | 3 (5.3) |
| Italy | 3 (5.3) |
| Australia | 2 (3.5) |
| Belgium | 2 (3.5) |
| Bulgaria | 2 (3.5) |
| Germany | 2 (3.5) |
| Indonesia | 2 (3.5) |
| Argentina | 1 (1.8) |
| Bangladesh | 1 (1.8) |
| Brazil | 1 (1.8) |
| Colombia | 1 (1.8) |
| Denmark | 1 (1.8) |
| Egypt | 1 (1.8) |
| France | 1 (1.8) |
| Ireland | 1 (1.8) |
| Japan | 1 (1.8) |
| North Macedonia | 1 (1.8) |
| Norway | 1 (1.8) |
| Philippines | 1 (1.8) |
| Poland | 1 (1.8) |
| Portugal | 1 (1.8) |
| Serbia | 1 (1.8) |
| Spain | 1 (1.8) |
| Sweden | 1 (1.8) |
| Switzerland | 1 (1.8) |
| Turkey | 1 (1.8) |
