# Supplementary material for: Evaluating International Diagnostic, Screening, and Monitoring Practices for Craniofacial Microsomia and Microtia: A Survey Study
Source: Cleft Palate Craniofac J. 2022 Apr 26;60(9):1118–27. doi: 10.1177/10556656221093912 (PMC10466995; doi:10.1177/10556656221093912)
Supplement: sj-docx-3-cpc-10.1177_10556656221093912 - Supplemental material for Evaluating International Diagnostic, Screening, and Monitoring Practices for Craniofacial Microsomia and Microtia: A Survey Study [file sj-docx-3-cpc-10.1177_10556656221093912.docx]

**Supplementary table 2: Qualitative comments**

| **Question number and summary** | **Comment summary** | **Comment details** |
| --- | --- | --- |
| 5. ICHOM criteria for CFM * (3.1) | Disagreement with criteria | Three minor criteria should be accompanied by one major criterion. |
|  |  | The third variant of the definition should include at least one craniofacial underdevelopment aspect. For example: pre-auricular tag and hemivertebrae and clefting is not "microsomia". |
|  |  | Unilateral congenital mandibular hypoplasia should suffice. |
|  |  | I would add Asymmetric facial movement to the minor criteria group. |
|  | Disagreement with inclusion of Goldenhar syndrome | [These criteria] make it very unclear what the relation is to Goldenhar syndrome |
|  |  | Epibulbar dermoids and hemivertebrae are features of Goldenhar syndrome which is a subset of CFM. |
|  | Neither agreement nor disagreement with criteria | [These criteria] are a useful option, but there are others as well. |
|  |  | In my opinion CFM must include mandibular hypoplasia. Three minor criteria only is not enough. |
|  | Agreement with criteria, disagreement with name of the condition | We do not agree that the condition should only be called craniofacial microsomia. In most cases, hemifacial microsomia is more appropriate. |
| 6. Isolated microtia is a mild form of CFM | Disagreement with statement | For diagnosis we need at least another anomaly. |
|  |  | If [there are] no other items, it is microtia solely. |
|  |  | Microtia is a single major criterion and not sufficient for diagnosis |
|  |  | See 5: if one uses the ICHOM criteria, it does not qualify. |
|  |  | Definitely not enough. |
|  |  | [Microtia] is an isolated form. So not a mild form. It is standing on its own, or it belongs to CFM. |
|  |  | Not if the face is symmetric. |
|  |  | Does not fulfill the criteria, although it is part of the phenotypic spectrum. |
|  |  | Could be, but I think that it is important to define the whether there is or not an involvement of the adjacent cranial bone or there is an isolated involvement of the external ear (not affecting the auditory channel and/or the temporal bone). |
|  |  | We consider this a specific entity. |
|  |  | Can be associated with CFM. |
|  |  | I believe this is unknown. |
|  |  | Clinically different. |
|  | Neither agreement nor disagreement with statement | Isolated microtia can be a mild form. However, we don’t know since no genetic cause can be proven. |
|  | Agreement with statement, additional comment: | All patients with microtia have some degree of ipsilateral soft palate dysfunction. |
|  | Other comments: | [Microtia] surgery is challenging. |
|  |  | Microtia is the most difficult challenge associated with CFM; even i admit as a [maxillofacial] surgeon we don't do ear helix reconstruction (we refer to ENT or Plastic colleagues) but when a patient present with sever mandibular hypoplasia and normal ear it is much easier to treat compared to patient with normal mandible and defective ear |
| 7. Other classification system used. | Other classification system: | Pruzansky. |
|  |  | Pruzansky classification for mandibular hypoplasia. |
|  |  | SAT [Skeletal, Auricular and Soft Tissue]. |
|  |  | Australian. |
|  |  | A phenotypic assessment similar to OMENS, but more descriptive. |
|  |  | HEAR MAPS [Hearing, Ear (microtia), Atresia grade, Remnant earlobe, Mandible development, Asymmetry of soft tissue, Paralysis of the facial nerve and Syndromes]. |
|  |  | We use Prada´s classification for diagnosis and treatment. |
|  | No classification system | I do not routinely use any classification system as it doesn’t affect my treatment plan for my patients. |
|  |  | No classification. |
|  | Unknown to respondent | The plastic surgeon of our team and the geneticist classify the patient. |
| 8. Timing of definitive classification | After multidisciplinary evaluation | After orthopantomogram/computed tomography answer and after results from echocardiogram/abdominal ultrasound/cervical spine examination or other specific examinations. |
|  |  | After the multidisciplinary team session |
|  |  | After careful and complete evaluation: pediatric, radiologic, genetics etc. |
|  |  | After the most invasive diagnostic that is done in the individual case. |
|  |  | In clinical genetics, we usually combine all the information we have from the complementary investigations. |
|  |  | After full radiographic and clinical assessment by the whole team has been carried out. |
|  |  | By multidisciplinary consultation |
|  | No classification used | I do not routinely use any classification system as it doesn’t affect my treatment plan for my patients. |
|  |  | No classification. |
|  | Unknown to respondent |  |
|  | Other comments: | If the sign is obvious [or the] mandible is very hypoplastic, the diagnosis can be [made] at first consultation. |
| 9. Preferred age/time for CT imaging | Based on clinical indication | If the scan has clinical implications. Pre-middle ear surgery (age 10) |
|  |  | Only when [scanning] influences decision making |
|  | Other comments: | I do not have an opinion about the timing, but I agree that it is important in order to assess the extent of the craniofacial involvement. |
| 10. Other structures evaluated through CT imaging (n=57) | Multiple structures | The entire facial skeleton |
|  |  | CT is often required for the middle ear but it is a waste of effort to then exclude other areas emphasizing the important of multispecialty collaboration. We have a specialist multidisciplinary clinic for HFM patients alone (which evolved out of a paediatric craniofacial clinic) and a separate clinic for microtia |
|  |  | Once we do a CT we tend to ask for all of the above. |
|  | Vertebrae in case visible. | |
|  | Vertebrae. | |
|  | Facial soft tissue. | |
|  | At times I use the CT to image the normal ear. | |
| 11. Biannual screening for obstructive sleep apnea at least until age of 6 years * (4.1.2) | Disagreement with indication | Depending on the clinical symptoms. |
|  |  | Depending on the degree of CFM. |
|  |  | Only if there is a strong clinical suspicion. OSA in unilateral CFM is uncommon and more prevalent in the severely affected. |
|  |  | Our experience of around 150 cases is that OSA is rare. |
|  |  | Screening only in patients with initial obstructive sleep apnea. |
|  |  | Only in severe cases. |
|  |  | We have to do only what is needed. |
|  |  | We managed more than 10 patients in last 5 years none of them suffered from OSA. |
|  | Disagreement with frequency | Depending of the child, once a year. |
|  |  | Biannually in first two years of life, then annually. |
|  |  | Not biannually in mild cases: then every three years |
|  |  | Needs assessment at first consultation of the patient. |
|  |  | Most children with CFM do not have OSA. Once it is excluded in infancy it is then checked only yearly. |
|  | Disagreement with use of questionnaire | We take history rather than use questionnaire. |
|  |  | They require a sleep study. A questionnaire alone is very unreliable and will not diagnose central sleep apnea which is frequently found in these infants. |
|  | Neither agree nor disagree | I actually screen all patients in my practice for this since it would be contraindicated for any patient to have outpatient surgery with OSA. |
| 12. Polysomnography in case of OSA suspicion based on questionnaire * (4.1.2) | Disagreement with indication | [Polysomnography] should be performed. |
|  |  | [Polysomnography is] difficult to do it in very young patients. |
|  | Neither agreement nor disagreement | In principle agree, but there should be clinical signs and complaints that fit in with the questionnaire results. Just a question of the wording of this question. |
| 13. Polysomnography for all Pruzansky-Kaban IIa or III or bilaterally affected mandibles * (4.1.2) | Disagreement with indication | As usual, you should have a firm indication to do this. |
|  |  | If symptomatic. |
|  |  | Only if there is complaint. |
|  |  | Most yes, all no because sometimes the bilateral case is only just bilateral and not severely so. |
|  |  | Only cases with clinically suspected sleep apnea, no CT scan in infants. |
|  |  | Do it on a case to case basis. |
|  |  | Only if symptoms. |
|  |  | Polysomnography based on history. |
|  |  | May or may not be indicated depending on clinical symptoms. |
|  |  | Depending on the patients: sometimes clinically obvious hence polysomnography is bypassed. |
|  | Disagreement on diagnostic course | Referral to sleep physician. |
|  |  | We would have patient evaluated by ENT and possible pneumogram first ENT exam would include direct visualization of the upper airway and larynx. |
| 14. Biannual screening for feeding difficulties at least until 6 years* (4.2.2) | Disagreement with indication | Not for mild cases. |
|  |  | The spectrum is very wide not everyone needs this. Which is also why the diagnostic tool is so strange. |
|  |  | Feeding issues do not seem to be a major issue, whereas psychological issues are - mandatory psychology screening not by a questionnaire is desirable in all cases. |
|  |  | If clinically relevant and depending on degree. |
|  | Disagreement with use of questionnaire | All children should be monitored for feeding difficulties, but without a questionnaire. |
|  |  | Both height and weight with percentiles should be performed at every visit. This is a much better indicator of feeding issues. |
|  |  | Unclear if a validated questionnaire exists, I would opt for pediatric ENT assessment. |
|  |  | Checked [based on history] during follow-up clinic. |
|  | Disagreement with frequency | Biannually in first two years of life, then annually |
|  |  | Annually may be sufficient |
|  |  | Feeding difficulties are rare. Infant screening is fundamental. |
| 15. Screening for preverbal communication at age 9 months * (4.3.2) | Disagreement on indication | This depends on whether a cleft or cleft related problem exist and movement of the lower jaw is hampered. However, a lot of the patients in the spectrum do not show that. |
|  |  | Depending on other clinical manifestations and diagnostic exams. |
|  |  | We individualize this topic. |
|  |  | Desirable but not essential in all cases. |
|  |  | It is uncommon to see verbal communication skills with CFM patients. |
|  |  | There are many other co-factor to evaluate |
|  | Disagreement on timing | At age of 12-18 months in our center. |
|  |  | Twelve months is more sensible as it fits better with clinic visit times. |
|  |  | At one year [and at] two years with phonatory guidance |
|  |  | Interventions may be needed after two years except date emergency. |
|  |  | Even earlier. |
|  |  | Too early. |
|  |  | Later assessment |
|  | Neither agreement nor disagreement | We usually don't screen [for] preverbal communication. |
|  |  | What do you mean about intervention? |
| 16. Biannual language evaluation between 2 and 8 years * (4.3.2) | Disagreement with frequency | Annually could be fine. Depending on type and degree of hearing loss. |
|  |  | At two [years], then annually. |
|  |  | At the age of 2-3 years and only repeated if issues |
|  |  | At one year [and at] two years with phonatory guidance. |
|  |  | If receptive and expressive skills are screened and OK, is there a prediction that this would change? |
|  | Disagreement with indication | This depends on whether a cleft or cleft related problem exist and movement of the lower jaw is hampered. However, a lot of the patients in the spectrum do not show that. |
|  |  | We individualize this. |
|  |  | Agree on the need for speech and language screening but only as proposed by the speech and language therapist. |
|  |  | It is uncommon to see language skill deficit with CFM. |
|  | Comment on evaluating discipline | By pediatrician or craniofacial team |
|  |  | Every single case must be evaluated by a dedicated [speech and language therapist]. |
| 17. In case of cleft palate: annual screening between 2 and 5 years by specialized speech and language therapist and adherence to cleft palate protocol * (4.3.2) | Disagreement with frequency | They should be followed according to a cleft protocol and in case needed more often. |
|  |  | Patients should begin speech screening by 9 months and be screened at 18 months and at two years to assure proper development of language and emergence of articulation. |
|  |  | At one year [and at] two years with phonatory guidance, then annually. |
|  | Comment on evaluating discipline | These cases are followed up in craniofacial team AND in cleft team, including orthodontist, speech therapist etc. |
|  |  | Every single case must be evaluated by a dedicated [speech and language therapist]. |
| 18. In case of cleft palate: velopharyngeal dysfunction assessment at 2 years or emergence of verbal output * (4.3.2) | Disagreement with timing | Velopharyngeal dysfunction should be assessed at the age of one year. |
|  |  | A three years. |
|  |  | At four years old. |
|  |  | We usually do that at the age of three to four. |
|  |  | Annually in the first two year after speech therapy. |
|  |  | Since palate surgery here [is] usually later ([at] about 2 years old or more), about 3 years old or about 6 months after surgery is best time to assess velopharyngeal dysfunction. |
|  | Disagreement with indication | Only if the child presents with articulation problems. |
|  |  | We had velopharyngeal dysfunction with patients suffering from both CFM and cleft palate. Not a common affection for CFM patients. |
|  | Agreement with statement, additional comment: | May be later depending on the child. |
|  |  | This is a very early assessment that might be repeated later depending on the development of the child before leading to consequences. |
|  | Other comments: | Velopharyngeal dysfunction assessment should be part of any speech and language therapy assessment. |
| 19. Velopharyngeal dysfunction screening at age 2 years in all patients* (4.3.2) | Disagreement with indication | Only if articulation problems arise. |
|  |  | We individualize this topic, and do no screening of asymptomatic cases. |
|  |  | Not necessarily and dependent on individual case and clinical evaluation. |
|  |  | Based on sign and symptoms. |
|  |  | Rarely a problem. |
|  |  | Not common at all to see velopharyngeal dysfunction in isolated CFM without cleft palate. |
|  | Disagreement with timing | Too early. |
|  |  | A bit later: three to four [years]. |
|  | Agreement with statement, additional comment: | We would suggest monitoring the speech and language development in order to be ready to start at the right moment a speech therapy when and if it is needed. |
|  |  | May be later depending on the child. |
|  | Neither agreement nor disagreement | Also evaluate for submucous clefts |
| 20. Neonatal hearing test in all patients * (4.4.2) | Disagreement with indication | All newborns should have hearing test including children without craniofacial microsomia. |
|  |  | A newborn hearing test for ALL newborns is standard in the UK within 10 days of birth. |
|  | Disagreement with timing | Can be performed at two to three months |
|  |  | Not neonatal, but babies |
|  | Agreement with statement, additional comment: | Absolutely! |
| 21. Complete audiological evaluation before age 3 months (if indicated) * (4.4.2) | Disagreement with timing | Too soon. |
| 22. Independent hearing assessment next to any national assessment | Disagreement | It should be a part of the work up by the craniofacial team. |
|  |  | Screening tests should be sufficient if hearing is normal. |
|  |  | No need for independent hearing assessment |
|  |  | Often an overtreatment. |
|  | Neither agree nor disagree | Hearing are included in national screening tests, and is carefully followed up if initial tests are failed. |
|  |  | Could be combined but mostly more are indicated. |
|  |  | in the Netherlands we have a national screening test, and because of the hearing problems, associated with CFM, they are at risk and will be checked regularly in the first year. |
|  | Agreement with statement, additional comment: | Both national and independent. |
| 23. Screening by orthoptist and ophthalmologist before age 5 years * (4.5.2) | Disagreement with indication | Only if there is ocular pathology. |
|  |  | Case-based decision |
|  |  | I don’t think it has relation. |
|  | Agreement with statement, additional comment: | I am not sure how mandatory that should be in the absence of any clinical signs or symptoms. |
| 24. Referral to ophthalmologist in case of lagophthalmos * (5.2.1) | Disagreement with indication | Only if there are symptoms. |
|  | Disagreement with discipline | Maxillofacial can do [this assessment]. |
|  |  | Usually our craniofacial staff can do this evaluation. |
|  | Agreement with statement, additional comment: | Should also be seen by an (oculo)plastic surgeon |
| 25. Cleft Q appearance for assessment of facial movement at ages 8, 12 and 22 years * (5.2.1) | Disagreement with indication | Individualized. |
|  |  | If initially present, further follow-up, otherwise not. |
|  |  | Case-dependent (family consent). |
|  |  | Only in rare cases with facial nerve [pathology], not routinely. |
|  | Disagreement with frequency | Follow-up in cases where initially present; no follow up in asymptomatic patients. |
|  | Disagreement with questionnaire | Fees to be paid. |
|  |  | I am not familiar with [the Cleft Q]. and don’t treat clefts anymore. |
|  |  | Not all centers use [the Cleft Q]. |
|  |  | I would need to know more about [the Cleft Q] before agreeing to such mandatory assessment. |
| 26. Screening by orthodontist from age 5 years for dental deformities * (4.6.2) | Disagreement with timing | At seven for orthodontic assessment and dental assessment age five [years]. |
|  |  | As soon as possible for intake. Indeed, at five years after intake and onwards |
|  | Neither agreement nor disagreement with statement | Our team evaluates dental development before age five. The pediatric dentistry evaluates when the temporary dentition is present in mouth. |
| 27. Orthodontic records at ages 6, 9, 12, 15 and 18 years * (4.6.2) | Disagreement with frequency | Our chronology recommends evaluation at the age of eight [years] and depending on the treatment options the other records will take place when needed. |
|  |  | It depends on individual dental development. |
|  |  | Variable ages based on dental development for individual child. |
|  |  | I am not at all sure that such structured invasive investigations are desirable and would like to see the supporting evidence for this. |
|  |  | At five, nine, 12, 15 and 18 years. |
|  |  | At five, 10, 15 and 18 years |
|  |  | We use five-six, 10 and 16 years and at the end of growth. |
|  |  | Under six [years] also. |
|  |  | At age 8 years; other records when needed depending on the treatment options |
|  |  | If there are important changes, even more often. |
|  |  | Records due to cost are taken as needed to begin orthopedic treatment or commence with surgery. |
|  |  | Agree with standardized time of assessment but I defer to my orthodontist to make that decision. |
|  |  | Based on orthodontist need. this number of records may be exaggerated |
| 28. Screening for neck/back symptoms during initial consultation and preoperatively * (4.7.2) | Disagreement with frequency | Necessary for preoperative workup, not during initial consultation if without symptoms. |
|  |  | Initial consultation often as a baby so symptoms will be impossible to elicit. |
|  | Disagreement with indication | Case-dependent |
|  | Agreement with statement, with additional comment. | In order to diagnose associated syndromes. |
|  |  | I would go much further. The incidence of spinal anomalies in HFM is very high. All children diagnosed with HFM should be seen by a pediatric orthopedic surgeon in their first few months and have at least plain x-rays. Any with abnormal findings should then be seen by a pediatric spinal surgeon. |
| 29. Neurological evaluation (ASAP) in case of neurologic symptoms (neck/back) * (4.7.2) | Disagreement with indication | On request by the pediatrician. |
|  |  | Not necessarily, unless clinically indicated. |
|  | Misinterpretation of the statement | Facial nerve weakness is very common but does not warrant evaluation by a pediatric neurologist [removed as the question pertains to neck/back -related neurological symptoms and not facial nerve weakness]. |
| 30. Spine radiography for vertebral screening in all patients | Disagreement with indication | Only if physical exam suggests abnormality. |
|  |  | Only if patient has difficulties or has an interest of this examination. |
|  |  | If indicated by history or exam. |
|  |  | If there is any suspicion |
|  |  | Depending on clinical symptoms. |
|  |  | In severe cases and if symptoms. |
|  |  | Only if symptoms are present. |
|  |  | Not if ok without any issues. |
|  |  | Maybe for mild CFM and without any symptoms, spine radiography is not necessary. |
|  |  | There is no evidence, however should seriously be considered in my opinion based on the high incidence and consequences of certain anomalies |
|  | Neither agreement nor disagreement with statement | Dependent on child |
|  |  | Should be done if symptomatic or before general anesthesia for surgery. |
|  | Agreement with statement, additional comment: | Especially if Goldenhar syndrome is suspected. |
|  |  | I would go much further. The incidence of spinal anomalies in HFM is very high. All children diagnosed with HFM should be seen by a pediatric orthopedic surgeon in their first few months and have at least plain x-rays. Any with abnormal findings should then be seen by a pediatric spinal surgeon. |
| 31. Age/time for vertebral screening (n=55) | Only when indicated | If patient has clinical problems. |
|  |  | As early as indicated. |
|  |  | If there are symptoms. |
|  |  | Depending on clinical symptoms. |
|  |  | [If] patient complains of backache or other obvious deformity. |
|  |  | Only with clinical examination. |
|  |  | When symptoms are present. |
|  |  | At initial consultation and again as soon as there is any suspicion of clinical relevance. |
|  |  | If clinical signs are present |
|  | Other comment: | Although not strictly recommended, children below 8 [years] are more susceptible to craniocervical injuries. |
|  |  | I am not sure when the best age to screen is, but I would follow accepted guidelines. |
| 32. Echocardiography for cardiac screening in all patients | Disagreement with indication | If indicated. |
|  |  | Depending on clinical symptoms. |
|  |  | Not sure if all patients require this. |
|  |  | Only in cases with symptoms. |
|  |  | Only if there is a murmur. |
|  |  | Based on cardiologist examination. |
|  |  | Only when this is necessary, and if there is a history of cardiac problems. |
|  |  | Depending of the status. |
|  |  | However, consultation to a pediatrician is indicated. |
|  | Neither agreement nor disagreement with statement | Only if indicated. |
|  |  | If there is any hint at cardiac anomalies (prenatal US, clinical). |
|  |  | Dependent on [the] individual. |
|  | Agreement with statement, additional comment: | As part of the diagnostic work-up of a syndromal case with possibly multiorgan involvement. |
|  |  | Absolutely. |
| 33. Age/time for cardiac screening (n=55) | Only when indicated | Depending on clinical symptoms. |
|  |  | Including in the pediatric examination. |
|  |  | Only in cases with symptoms. |
|  |  | If there is any hint at cardiac anomalies (prenatal US, clinical). |
|  |  | If there is a murmur. |
|  |  | If some symptoms is evident. |
|  | Before 1 year | But often we see these patients later on and they are referred then. |
| 34. Renal ultrasonography for renal screening in all patients | Disagreement with indication | If indicated. |
|  |  | If required. |
|  |  | Only with symptoms. |
|  |  | Only if clinically indicated. |
|  |  | Only if ear pathology is present. |
|  | Neither agreement nor disagreement with statement | If there is any hint at cardiac anomalies (prenatal US, clinical). |
|  |  | Dependent on [the] individual. |
|  | Agreement with statement, additional comment: | As part of the diagnostic work-up of a syndromal case with possibly multiorgan involvement. |
|  |  | But the incidence of renal issues seems very rare in my practice so I am not sure how valuable this is. |
|  |  | However, this not included in the guideline. |
|  | Other comments: | Unsure if essential, but would do no harm. |
| 35. Age/time for renal screening (n=54) | Only when indicated | If patient complains of urological problems. |
|  |  | Only if symptoms are present. |
|  |  | Suspicion of renal issues. |
|  |  | Based on sign and symptoms. |
|  |  | If there is any hint at cardiac anomalies (prenatal US, clinical). |
|  | Before 1 year | Or when we see the patient for the first time. |
|  |  | [During] the pediatric examination. |
|  | Other comment: | Additional anomalies might deserve follow-up and treatment |
| 62. Screening for additional anomalies (not mentioned above) for all patients | Agreement with statement, additional anomalies to screen: | Head to toe examination. |
|  |  | These patients requires special analysis and detection of facial minor anomalies. |
|  |  | Psycho social issues and potential learning disability. |
|  |  | Genetic clinic consultation is done at our center. |
|  |  | Screening in the first year for age for all the known associations with HFM by a pediatrician is highly desirable. |
|  |  | Genetics, growth, hand/feet. |
|  |  | Geneticist screens all patients. |
|  |  | General pediatrician will review patients. |
|  |  | Full clinical examination |
|  |  | Epibulbar dermoids, preauricular tags or cysts, macrostomia. |
|  |  | Vertebral defects, anal atresia, cardiac defects, tracheo-esophageal fistula, renal anomalies and limb abnormalities (VACTERL). |
|  |  | Facial nerve pathology, contralateral ear function, genetics (if appropriate). |
|  |  | All patients should be referred to a clinical geneticist to look for underlying genetic disorders like EFTUD2 or spliceosomal defects. |
|  |  | Cardiac and spine anomalies are so common with CFM (early echocardiogram and lateral neck x-ray) |
|  | Neither agreement nor disagreement with statement | On indication: yes, but not always in each patient. |
|  |  | I think that the vertebral X-ray, hearing and ophthalmologic screening test, as well as the heart and abdominal ultrasound should be sufficient. |
|  | Disagreement with statement | Too many types of anomalies |
|  |  | As indicated based on clinical exam. |
| 37. Screening by clinical geneticist for all patients | Disagreement with indication | If the patient agrees and there is available clinical geneticist in the country. |
|  |  | Selected patients only. |
|  |  | May not be necessary. A pediatrician may suffice. |
|  | Neither agreement nor disagreement with statement | Not paid by insurance. |
|  | Agreement with statement, additional comment: | Especially in rare family cases or atypical cases. We genetically test more and more. |
|  |  | If available only, we do here in Egypt only with familial cases; because of high cost. But we wish to apply to every patient. |
| 38. Age/time for screening by clinical geneticist (n=54) | Only when indicated | Age would depend on child’s findings and symptoms. |
|  |  | When the patient or parents are ready for this. |
|  |  | Parents patients should be willing. They need to be counseled about the pros and cons. |
|  | Before 1 year of age | And at age of four. Before attending primary school |
| 39. Access to clinical psychology service (all patients) * (4.8.2) | Disagreement with indication | Only with an indication to do so. |
|  |  | Depending on clinical symptoms. |
|  |  | Evaluate case by case and family by family. |
|  |  | Only if needed. |
|  | Agreement with statement, additional comment: | But this is not organized in our team, unfortunately; individualized referral. |
| 40. Screening for psychological wellbeing at key life transitions * (4.8.2) | Disagreement with frequency | Depending on each patient. So much examination doesn’t allow the patient to feel normal. |
|  | Agreement with statement, additional comment: | This is executed by Center for rare diseases, in our craniofacial team. |
| 41. Routinely screening for psychosocial wellbeing and family stress using validated self-reported psychological outcome measures at ages 2, 5, 8 and 22 * (4.8.2) | Disagreement with questionnaires | The aforementioned scale/test are not validated in Italy. |
|  |  | Fees to be paid. |
|  |  | We don’t have this type of evaluation using these tools. |
|  |  | Due to costs. |
|  |  | We do not use CleftQ but screening for potential problems is done in each patient at every routine evaluation |
|  |  | Agree just add HearQL |
|  | Disagreement with indication | Only as a scientific study. |
|  |  | Depends on individual impact and conditions |
|  |  | This is a good guide to psychological assessments but should not be mandatory in all cases and all modalities. |
|  |  | As clinically indicated |
|  | Disagreement with timing | I do not understand why nothing exists between eight and 22. |
|  |  | With school entry and thereafter if indicated. |
|  |  | Our psychologist believes pre adolescence to be a fundamental time. |
|  | Agreement with statement, additional comment: | Agree with the concept of screening. Not sure which tests are best. |
|  |  | Depends on individual impact and conditions |
| 42. Disciplines needed in CFM team (6) | Other disciplines needed: | Pediatric dentist |
|  |  | Special care dentist |
|  |  | Dentist |
|  |  | Pediatric dentist. We have the support of other specialties in our hospital. |
|  |  | Specialist nurse |
|  |  | Pediatric dentist |
|  |  | Trained nursing staff. |
|  |  | General dentist |
|  |  | Orthoptist, neonatologist. |
|  |  | Pediatric dentist. |
|  |  | Occupational therapist. |
|  | Other comments: | Now I understand why you wish to call the condition CFM - so you can then lead on to say it can only be treated in a designated craniofacial center. |
|  |  | This [plastic surgeon, maxillofacial surgeon, ENT/audiology, psychology, orthodontics, pediatric anesthesiology, pediatrician, social worker and prosthetist] is the minimum requirement to treat more than 90% of cases. |
|  |  | The unchecked ones [ophthalmologist, pediatric anesthesiologist, pediatric intensivist, neurosurgeon or orthopedic surgeon, pediatric radiologist, pedagogical worker, (facial) physical therapist, prosthetist and respiratory team] would be a bonus but are not required. |
| 43. Same diagnostic and screening protocol for patients with isolated microtia | Disagreement with statement | If indicated. |
|  |  | Individual case analysis. |
|  |  | If only microtia, no need for CFM other extensive protocols as distraction osteogenesis of free flap reconstruction. Only (ENT or Plastic) team. |
|  |  | But [patients] should be properly informed about the consequences of this anomaly. |
|  |  | Selected cases. |
|  |  | As clinically indicated by a physical examination |
|  |  | Based on signs and symptoms |
|  |  | Once microtia is confirmed as isolated with no other anomalies, ongoing screening is not necessary. |
|  | Neither agreement nor disagreement with statement | Only if there are any additional anomalies or at least a suspicion. |
|  |  | Microtia without other anomalies doesn’t need [the same protocol]. |
|  |  | Don't know if they need the same, think a lot of the team is not needed. I should be selective, make a specific health-care path |
|  | Agreement with statement, additional comment: | Might be part of the same spectrum. Until you have not made an inventory of possible associated anomalies these might be missed. Furthermore, the psychological impact might be the same. |
|  |  | In order to be able to assert a microtia as "isolated", a multiorgan evaluation is required. |
|  |  | Otherwise you are not able to define it as isolated microtia. |
| 44. Additional comments | Degree of CFM varies so degree is important to define. | |
|  | Radiation considerations for radiological diagnostic examination. | |
|  | All this screening is good for scientific reasons, but I think all of this should be applied only in moderate/severe cases. | |
|  | This is a comprehensive review of this complex condition needing multi-modality treatment approach. I agree with most of the statements that comply with published guidelines. | |
|  | Patients need regular follow-up. | |
|  | I would suggest to consider the classification developed by Rolando Prada, from Bogotá, Colombia. | |
|  | Unfortunately, the guideline, as already mentioned to the authors, has some mistakes in terms of functional treatment of mandibular asymmetry and need for early distraction, both of which have shown very poor prognosis. | |
|  | Microtia can be part of CFM or as an isolated microtia. Tessier 7 cleft can be part of CFM, but not all type of facial clefting is CFM. | |
|  | Aesthetic corrections at completion of facial growth like fat grafting should also be included in the management plan. | |
|  | Each patient is unique. | |

* From European Guideline Craniofacial Microsomia (chapter)
