# Supplementary figures and images for: Evaluating International Diagnostic, Screening, and Monitoring Practices for Craniofacial Microsomia and Microtia: A Survey Study
Source: Cleft Palate Craniofac J. 2022 Apr 26;60(9):1118–27. doi: 10.1177/10556656221093912 (PMC10466995; doi:10.1177/10556656221093912)

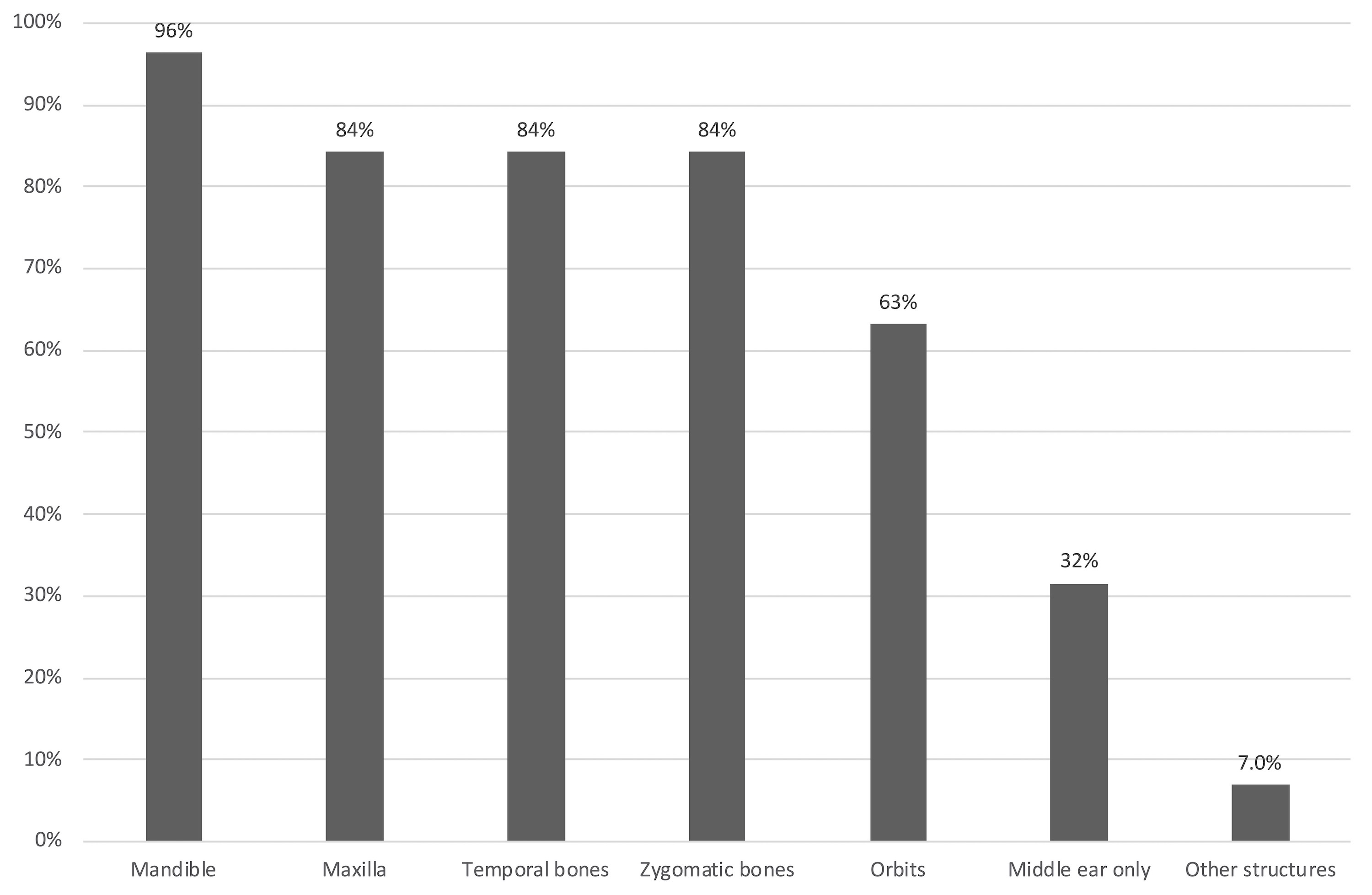

Supplement: sj-jpg-4-cpc-10.1177_10556656221093912 - Supplemental material for Evaluating International Diagnostic, Screening, and Monitoring Practices for Craniofacial Microsomia and Microtia: A Survey Study [file sj-jpg-4-cpc-10.1177_10556656221093912.jpg]

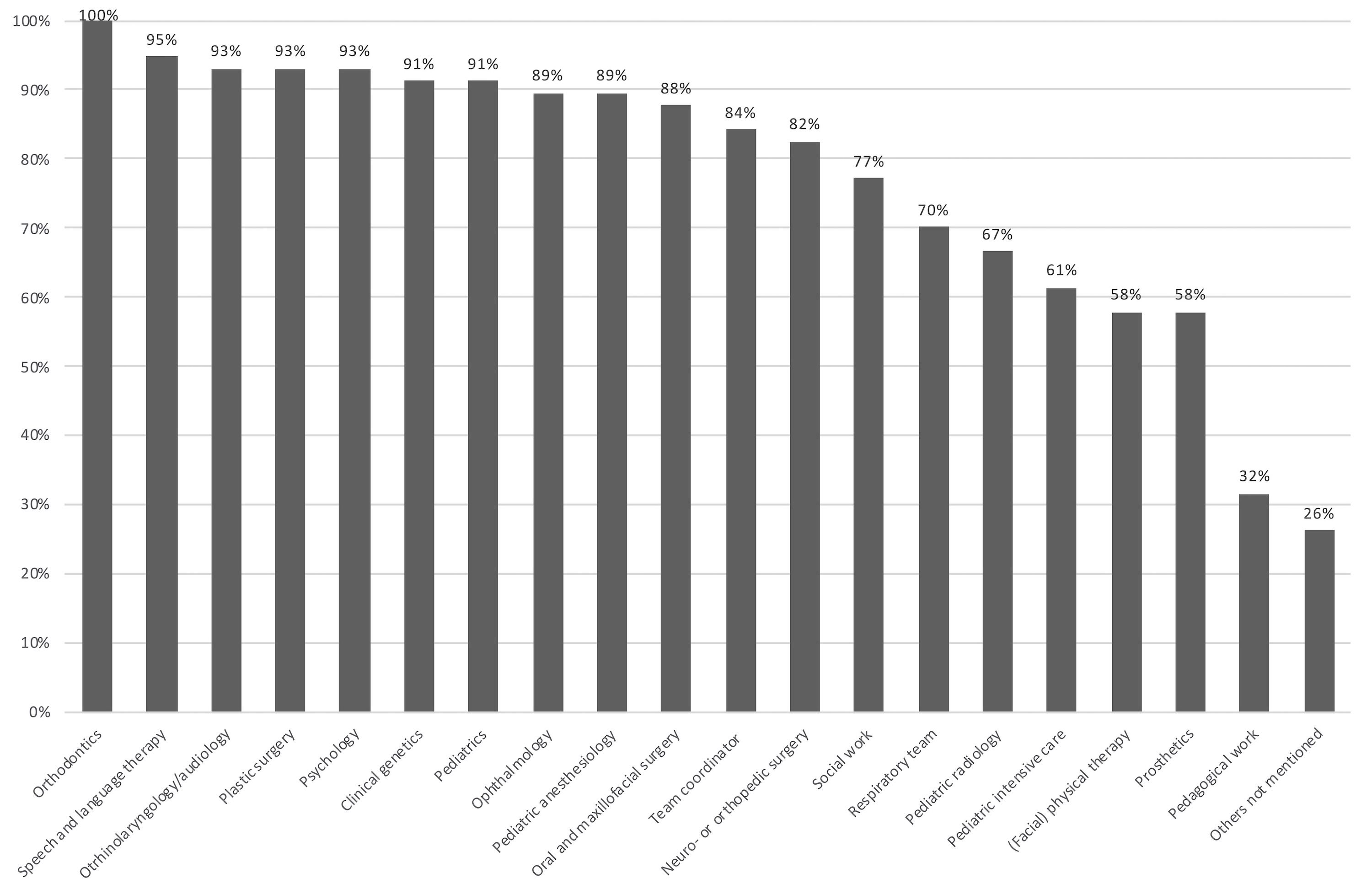

Supplement: sj-jpg-5-cpc-10.1177_10556656221093912 - Supplemental material for Evaluating International Diagnostic, Screening, and Monitoring Practices for Craniofacial Microsomia and Microtia: A Survey Study [file sj-jpg-5-cpc-10.1177_10556656221093912.jpg]
