# Supplementary material for: Evaluating International Diagnostic, Screening, and Monitoring Practices for Craniofacial Microsomia and Microtia: A Survey Study
Source: Cleft Palate Craniofac J. 2022 Apr 26;60(9):1118–27. doi: 10.1177/10556656221093912 (PMC10466995; doi:10.1177/10556656221093912)
Supplement: sj-docx-6-cpc-10.1177_10556656221093912 - Supplemental material for Evaluating International Diagnostic, Screening, and Monitoring Practices for Craniofacial Microsomia and Microtia: A Survey Study [file sj-docx-6-cpc-10.1177_10556656221093912.docx]

**Appendix B: survey questions**

Permission to republish statements from the European Guideline Craniofacial Microsomia^1^ has been granted by Wolters Kluwer Health, Inc.. Questions where multiple answers could be checked have been marked with an asterisk (*).

1. Hospital name
2. City
3. Country
4. Function *
   1. Plastic Surgeon
   2. Maxillofacial surgeon
   3. ENT surgeon
   4. Pediatrician
   5. Orthodontist
   6. Clinical Geneticist
   7. Physician Assistant
   8. Nurse Practitioner
   9. Other
5. Our center uses the following criteria for the diagnosis CFM (developed by the ICHOM Craniofacial Microsomia group) (European Guideline Craniofacial Microsomia Chapter 3.1)

| CFM is defined by: | 2 major criteria, or 1 major + 1 minor criteria, or 3+ minor criteria |
| --- | --- |
| Major criteria | Mandibular hypoplasia  Microtia  Orbital / facial bone hypoplasia  Asymmetric facial movement |
| Minor criteria | Facial soft tissue deficiency  Pre-auricular tags Macrostomia  Clefting  Epibulbar dermoids Hemivertebrae |

1. Isolated microtia is a mild form of CFM.
2. Which classification system do you use for classifying CFM*:
   1. The Orbit, Mandible, Ear, Nerve and Soft Tissue (O.M.E.N.S.)
   2. The Phenotypic Assessment Tool for Craniofacial Microsomia (PAT-CFM)
   3. Other
3. When do you definitively classify patients? *
   1. During the first consultation
   2. After CT scan
   3. After panoramic radiography
   4. Other
4. A CT scan (of the mandible and middle ear) should be performed*:
   1. Before the age of 1 year
   2. Before the age of 5 years
   3. As a diagnostic and planning tool for mandibular/maxillofacial surgery
   4. As a diagnostic and planning tool for ENT surgery
   5. Other
5. The CT scan should be used to evaluate at least*:
   1. Mandible
   2. Maxilla
   3. Zygoma
   4. Temporal bones (including the middle ear)
   5. Orbits
   6. Middle ear
   7. Other structures not mentioned above
6. All patients with craniofacial microsomia should be screened with a questionnaire biannually, at least up to the age of six, in the outpatient department for a clinical history of obstructive sleep apnoea (European Guideline Craniofacial Microsomia Chapter 4.1.2)
7. If there is a suspicion of obstructive sleep apnoea based on a questionnaire, a polysomnography (sleep study) has to be performed (European Guideline Craniofacial Microsomia Chapter 4.1.2).
8. All patients who have Pruzansky-Kaban IIb or III mandibles and/or are bilaterally affected have to undergo a polysomnography (sleep study) to screen for obstructive sleep apnoea in the first year of life (European Guideline Craniofacial Microsomia Chapter 4.1.2).
9. Children with craniofacial microsomia should be screened with a questionnaire biannually, at least up to the age of six, and monitored regularly for feeding difficulties by a paediatrician or multidisciplinary team (European Guideline Craniofacial Microsomia Chapter 4.2.2).
10. Screen preverbal communication and babbling skills at the age of nine months to decide if intervention is warranted (European Guideline Craniofacial Microsomia Chapter 4.3.2).
11. Evaluate receptive and expressive language skills at the age of two years and biannually until the age of eight years in all patients with craniofacial microsomia. (European Guideline Craniofacial Microsomia Chapter 4.3.2).
12. Children with craniofacial microsomia and associated cleft palate should be screened annually from 2-5 years by the Cleft-Craniofacial speech and language therapist and should follow the local Cleft Palate Protocol (European Guideline Craniofacial Microsomia Chapter 4.3.2).
13. Velopharyngeal dysfunction should be assessed at the age of two years or when verbal output has emerged (European Guideline Craniofacial Microsomia Chapter 4.3.2).
14. Children with craniofacial microsomia without cleft palate should also be screened at the age of two years to examine for potential risk of velopharyngeal dysfunction related to their asymmetrical structure. (European Guideline Craniofacial Microsomia Chapter 4.3.2).
15. Perform neonatal hearing test in all newborns with craniofacial microsomia (European Guideline Craniofacial Microsomia Chapter 4.4.2).
16. If indicated, complete audiological evaluation in an experienced audiology centre should be performed before the age of three months to ensure timely treatment (European Guideline Craniofacial Microsomia Chapter 4.4.2).
17. Hearing should be evaluated independently from national screening tests.
18. All patients with craniofacial microsomia should be screened at least once during the visual development (before the age of five) by an orthoptist and ophthalmologist (European Guideline Craniofacial Microsomia Chapter 4.5.2).
19. Refer all craniofacial microsomia patients with lagophthalmos to an ophthalmologist (European Guideline Craniofacial Microsomia Chapter 5.2.1).
20. Facial movement should by assessed with the CleftQ Appearance at age 8, 12, and 22 (European Guideline Craniofacial Microsomia Chapter 5.2.1).
21. Patients with craniofacial microsomia should be seen from age five by an orthodontist within a multidisciplinary team to diagnose dental deformities (European Guideline Craniofacial Microsomia Chapter 4.6.2).
22. Take orthodontic records in a structured schedule at 6, 9, 12, 15 and 18 years of age (European Guideline Craniofacial Microsomia Chapter 4.6.2).
23. Screening questions and clinical examinations related to neck/back symptoms should be undertaken at initial consultation and as part of preoperative workup (European Guideline Craniofacial Microsomia Chapter 4.7.2).
24. All patients with craniofacial microsomia who have neurologic symptoms (e.g. paraesthesia, numbness, or weakness) or neck pain suggestive of neuronal injury should be evaluated as soon as possible by a (paediatric) neurologist (European Guideline Craniofacial Microsomia Chapter 4.7.2).
25. All patients with CFM should be screened for vertebral anomalies using spine radiography.
26. Screening for vertebral anomalies should be done:
    1. Before the age of 1 year
    2. Before the age of 5 years
    3. Before the age of 10 years
    4. Only if indicated (please specify when screening is indicated in the comment box)
27. All patients with CFM should be screened for cardiac anomalies echocardiography.
28. Screening for cardiac anomalies should be done:
    1. Before the age of 1 year
    2. Before the age of 5 years
    3. Before the age of 10 years
    4. Only if indicated (please specify)
29. All patients with CFM should be screened for renal anomalies using ultrasonography.
30. Screening for renal anomalies should be done:
    1. Before the age of 1 year
    2. Before the age of 5 years
    3. Before the age of 10 years
    4. Only if indicated (please specify)
31. All patients should be screened for additional anomalies not yet mentioned.
32. All patients should be screened by a clinical geneticist.
33. Screening by the clinical geneticist should be done:
    1. Before the age of 1 year
    2. Before the age of 5 years
    3. Before the age of 10 years
    4. Only if indicated (please specify)
34. All craniofacial microsomia patients should have access to a clinical psychology service with appropriate professional expertise and knowledge of craniofacial microsomia (European Guideline Craniofacial Microsomia Chapter 4.8.2).
35. Time points for reviews and screening should observe key life transitions such as birth, starting school, transition to secondary school, etc. (European Guideline Craniofacial Microsomia Chapter 4.8.2).
36. To measure psychosocial wellbeing and family stress, validated self-reported psychological outcome measures should be obtained from all craniofacial microsomia patients as a matter of routine to screen for the presence of behavioural, emotional, social and/or learning difficulties. This includes the CleftQ, CFEQ, YP-CORE, HADS and Distress Thermometer for Parents and should be performed at age at ages 2, 5, 8 and 22 (European Guideline Craniofacial Microsomia Chapter 4.8.2).
37. A craniofacial centre should have the following care providers (European Guideline Craniofacial Microsomia Chapter 6)*.
    1. Maxillofacial surgeon
    2. Plastic surgeon
    3. ENTsurgeon/audiologist
    4. Psychologist
    5. Orthodontist
    6. Ophthalmologist
    7. Paediatric anaesthesiologists
    8. Team coordinator
    9. Paediatrician
    10. Clinical geneticist
    11. Paediatric intensivist
    12. Neurosurgeon and/or orthopaedic surgeon for spinal anomalies
    13. Paediatric radiologist
    14. Social worker
    15. Speech therapists
    16. Pedagogical worker
    17. (Facial) physical therapist
    18. Prosthetist
    19. Respiratory team
38. Patients with isolated microtia should follow the same diagnostic and screening protocol (as specified above) as patients with CFM.
39. Additional comments

**References**

1. Renkema, R. W. and the and the ERN CRANIO Working Group on Craniofacial Microsomia (2020). European Guideline Craniofacial Microsomia. *Journal of Craniofacial Surgery, 31*, 2385-2484. doi:10.1097/scs.0000000000006691
